# Predictive Metagenomic Profiling, Urine Metabolomics, and Human Marker Gene Expression as an Integrated Approach to Study Alopecia Areata

**DOI:** 10.3389/fcimb.2020.00146

**Published:** 2020-04-29

**Authors:** Daniela Pinto, Francesco Maria Calabrese, Maria De Angelis, Giuseppe Celano, Giammaria Giuliani, Marco Gobbetti, Fabio Rinaldi

**Affiliations:** ^1^Human Microbiome Advanced Project-HMPA, Giuliani SpA, Milan, Italy; ^2^Department of Soil, Plant, and Food Sciences, University of Bari Aldo Moro, Bari, Italy; ^3^Faculty of Science and Technology, Free University of Bozen-Bolzano, Bolzano, Italy

**Keywords:** alopecia areata, microbiome, KEGG, scalp microbiome, metabolic pathways, metagenomic profiles, VOCs, metabolomics

## Abstract

Involvement of the microbiome in many different scalp conditions has been investigated over the years. Studies on the role of the scalp microbiome in specific diseases, such as those involving hair growth alterations like non-cicatricial [androgenetic alopecia (AGA), alopecia areata (AA)] and cicatricial alopecia lichen planopilaris, are of major importance. In the present work, we highlighted the differences in microbial populations inhabiting the scalp of AA subjects and a healthy sample cohort by using an integrated approach relying on metagenomic targeted 16S sequencing analysis, urine metabolomics, and human marker gene expression. Significant differences in genera abundances (*p* < 0.05) were found in the hypodermis and especially the dermis layer. Based on 16S sequencing data, we explored the differences in predicted KEGG pathways and identified some significant differences in predicted pathways related to the AA pathologic condition such as flagellar, assembly, bacterial chemotaxis, mineral absorption, ABC transporters, cellular antigens, glycosaminoglycan degradation, lysosome, sphingolipid metabolism, cell division, protein digestion and absorption, and energy metabolism. All predicted pathways were significantly enhanced in AA samples compared to expression in healthy samples, with the exceptions of mineral absorption, and ABC transporters. We also determined the expression of *TNF-*α, *FAS, KCNA3, NOD-2*, and *SOD-2* genes and explored the relationships between human gene expression levels and microbiome composition by Pearson's correlation analysis; here, significant correlations both positive (SOD vs. *Staphylococcus, Candidatus Aquiluna*) and negative (*FAS* and *SOD2* vs. *Anaerococcus, Neisseria*, and *Acinetobacter*) were highlighted. Finally, we inspected volatile organic metabolite profiles in urinary samples and detected statistically significant differences (menthol, methanethiol, dihydrodehydro-beta-ionone, 2,5-dimethylfuran, 1,2,3,4, tetrahydro-1,5,7-trimethylnapthalene) when comparing AA and healthy subject groups. This multiple comparison approach highlighted potential traits associated with AA and their relationship with the microbiota inhabiting the scalp, opening up novel therapeutic interventions in such kind of hair growth disorders mainly by means of prebiotics, probiotics, and postbiotics.

## Introduction

Increasing evidence is helping to elucidate the role of the microbiome in human health and disease (Cho and Blaser, 2012). More recently, scientists have highlighted the crucial role of the microbiome in skin health (Grice, 2014), even though, at present, few studies have focused on hair growth-related conditions. In recent years, researchers' efforts have focused on understanding the unique relationship between the microbial communities inhabiting the human body, from the gut to the oral cavity and skin (Blum, 2017), and more recently, the scalp (Clavaud et al., 2013; Rinaldi et al., 2018; Saxena et al., 2018; Polak-Witka et al., 2020). As the largest organ of the human body, the skin comprises highly diversified ecosystems across different regions of the human body (Byrd et al., 2018; Langan et al., 2018), each of which is characterized by different water and sebum contents, pH values, temperatures, and moisture levels. Strong inter-individual diversity has also been reported (Nakatsuji et al., 2013; Oh et al., 2014).

Compared to other skin and associated ecosystems, the scalp is thicker, more vascularized, characterized by the presence of more sebaceous glands, and has a more acidic pH. For this reason, the scalp microbial population is of particular interest (Rinaldi et al., 2018). The scalp is inhabited mainly by bacteria belonging to *Propionibacterium* and *Staphylococcus* genera (Pinto et al., 2019) and fungi such as *Malassezia* spp. (Clavaud et al., 2013), as well as other microorganisms such as mites or viruses (Boxman et al., 1997; Scharschmidt, 2017). All of these microbial populations cooperate to create a specific ecosystem that can influence the health and functionality of the scalp and hair follicles (Zeeuwen et al., 2012; Polak-Witka et al., 2020).

The involvement of the microbiome in many different scalp conditions has been investigated over the years. Larger studies have been related to dandruff (Clavaud et al., 2013; Xu et al., 2016; Saxena et al., 2018) and seborrheic dermatitis (Park et al., 2012; Soares et al., 2016), whereas other studies have dealt with folliculitis decalvans (Prohic, 2003; Takahata et al., 2007; Zomorodian et al., 2008; Matard et al., 2013; Gomez-Moyano et al., 2014; Rudramurthy et al., 2014). Only recently has the attention of researchers also focused on scalp conditions related to hair growth such as androgenetic alopecia (AGA) (Mahé et al., 2000; Rinaldi et al., 2018) and alopecia areata (AA) (Rinaldi et al., 2018; Ho et al., 2019; Pinto et al., 2019).

Among hair growth diseases, AA is the second most common (Odom et al., 2006), and based on its etiology, the contribution of autoimmunity has been well-established (McElwee et al., 2013). The psychological impact of this disorder cannot be neglected; the unpredictable and variable pathology course often results in psychological consequences for patients that typically experienced the distress symptomatology (Davey et al., 2019). However, therapy for AA remains a challenge. Among proposed causes, a link with the gut microbiome has also been hypothesized (Rebello et al., 2017; Borde and Åstrand, 2018). In a recently published clinical investigation, we reported a bacterial shift in subjects affected by AA (Pinto et al., 2019). Microbial populations inhabiting a given ecosystem entirely represent a complex community not only in terms of composition but also in terms of functionality. For this reason, the microbiome signature in the scalp can no longer be neglected. The study of the role of the scalp microbiome in specific diseases such as those involving hair growth alterations like non-cicatricial (AGA, AA) and cicatricial alopecia lichen planopilaris is of major importance.

Metabolites and small molecules mediate the relationship between the host and microbiota and represent a key point in the study of microbiota-induced changes to host physiology and their involvement in disease development. Thus, upon considering the role of micro- and macronutrients in hair physiology, it is necessary to investigate the impact of the microbial population and associated metabolites. The application of “omics” technologies to the study metagenomics provides a powerful tool to understand the molecular host–microbial causal relationships underlying the contribution of bacteria to pathologic conditions (Sharon et al., 2014). This study aimed to identify distinct scalp metagenomic profiles as well as patterns of scalp marker gene and urine metabolite expression. We report here the taxonomic classification of microbe ecology, with KEGG-based pathway prediction, comparing individuals affected by AA and a group of healthy subjects. The current study provides new perspectives for a deeper understanding of the involvement of the microbiome in scalp pathologic conditions related to hair growth, both in terms of composition and functionality.

## Materials and Methods

### Subject Recruitment

A cohort of 47 healthy and AA subjects (ages ranging from 20 to 60 years and a proportion of males equal to 40%) was enrolled under dermatological control at the private Italian dermatological clinic Studio Rinaldi (Milan, Italy). For each AA patient, essential background data were collected as a baseline according to the guidelines of the National Alopecia Areata Foundation (Olsen et al., 2004). Healthy subjects were enrolled following clinical examination and in the absence of any history of dermatological or scalp disorders. The inclusion criteria were as follows: male and female 20 to 60 years old; AA with a SALT (Severity of Alopecia Tool) score from S2 to S5; subjects agreed to follow the instructions they received from the investigator and were able to return to the study center at the established times; subjects agreed to not receive any drugs/cosmetics treatments that could interfere with the study results; no participation in a similar study actually or during the previous 6 months; not pregnant or breastfeeding; no antibiotic use in the last 30 days before sampling; no probiotic use in the last 15 days; last shampoo performed 48 h before sampling; not suffering from other dermatological diseases; no anti-tumor, immunosuppressant, or radiation therapy in the last 3 months; and no topical or hormonal therapy on the scalp in the last 3 months. Subjects also agreed to sign the informed consent form. The study was performed under the approval of the Ethical Independent Committee for Clinical, not pharmacological investigation in Genoa (Italy), and all patients were evaluated and enrolled after signing informed consent.

### Collection of Samples

Some enrolled subjects were already analyzed in a previously published cohort (Pinto et al., 2019), and here, the scalp microbiome and metabolites in urinary samples were assessed. Scalp samples were collected with a swab procedure according to previously reported methods. For each subject, an area of 16 cm^2^ was sampled. After collection, samples were stored at 4°C until DNA extraction. Four control and four AA were sampled to assess the microbial community in the subepidermal compartments of the scalp based on a 4-mm biopsy punch, as described by Pinto et al. (2019).

### Microbial DNA Extraction

DNA extraction from the swab and the three scalp layers (deep epidermis, dermis, and hypodermis biopsies) was performed using a QIAamp UCP Pathogen Mini Kit (Qiagen, Milan, Italy) according to the manufacturer's protocol, with minor modifications (Gao et al., 2010). The DNeasy Tissue Kit (Qiagen) was used for biopsy samples. Extracted DNA was finally suspended in DNAse-free water and quantified with the QIAxpert system (Qiagen) before high throughput 16S sequencing.

### High Throughput 16S Sequencing

DNA samples were amplified according to Caporaso et al. (2011) and Kozich et al. (2013) for the variable region V3-V4 using the universal prokaryotic primers 341 F CTGNCAGCMGCCGCGGTAA (Takahashi et al., 2014; Apprill et al., 2015) and 806bR GGACTACNVGGGTWTCTAAT (Parada et al., 2016; Walters et al., 2016). Illumina MiSeq V3 and V4 sequencing was carried out at Personal Genomics, Verona, Italy. High-fidelity polymerase (AccuStart II PCR ToughMix, Quantabio, Beverly, MA) was used for amplicon generation. The resultant amplicons were normalized using a SequalPrep Plate Normalization Kit (ThermoFisher Scientific, Monza, Italy) and the final concentration of the library was determined using a fluorometric kit (Qubit, Life technologies, Carlsbad, CA, USA). Obtained libraries were mixed with PhiX control libraries (Illumina-generated). Runs were performed using Real-Time Analysis software (RTA) v. 1.16.18 and 1.17.22 and MiSeq Control Software (MCS) v. 2.0.5 and 2.1.13. Four sequencing runs were performed with RTA v. 1.18.54, MCS v. 2.6, based on a target of 25% PhiX and 600–700 k/mm^2^ cluster densities according to Illumina specifications. Basecalls from Illumina High Throughput Sequencing (HTS) machines were converted to fastQ files using bcl2fastq (Illumina) software v2.20.0.42, and quality control was carried out with the bcl2fastq (Illumina) utility. FastQC v0.11.5. was used for quality control of fastq reads. Cutadapt v. 1.14 (Martin, 2011) and Sickle v. 1.33 (Joshi and Fass, 2011) toolkits were used for the quality trimming of primers and adaptors, respectively. Finally, paired-end reads were assembled using Pandaseq v. 2.11 (Masella et al., 2012), and clustering was carried out using closed-reference OTU picking and the *de novo* OTU picking protocol of QIIME v1.9 (Bolyen et al., 2019). The Greengenes database v13_8 was used as a reference for bacterial taxonomic assignment (Desantis et al., 2006). Amplicon reads were also analyzed to inspect alpha diversity based on the Shannon index using QIIME v1.9.

### Predictive Functional Profiling of Microbial Communities

Based on bacterial 16S rRNA gene sequences, the metabolic functions of the scalp microbiome were predicted using Phylogenetic Investigation of Communities by Reconstruction of Unobserved States (PICRUSt) version 1.1.4 (Langille et al., 2013) and the metagenomic-based analysis Kyoto Encyclopedia of Genes and Genomes (KEGG). First, a BIOM-formatted OTU table was generated using the make.biom command of the Mothur program based on a Greengenes database (May 2013 ver.; http://greengenes.lbl.gov). The abundance of each OTU was corrected to reflect the true bacterial abundance by normalizing the 16S rRNA copy number for each OTU. KEGG orthology abundances for a given OTU, table-picked against the newest version of Greengenes database, were calculated by locally running the Picrust “predict_metagenomes.py” script.

The accuracy of the predicted metagenomes was determined by the NSTI (nearest sequenced taxon index). NSTI quantifies the availability of nearby representative genomes for each microbiome sample and was determined as the sum of phylogenetic distances from each microbe in the OTU table to its adjacent relative with a sequenced reference genome. Low NSTI values indicate better accuracy in metagenome prediction. The gene functions classified by KO were further categorized into KEGG pathways using the “categorize_by_function.py” PICRUSt script, which collapses thousands of predicted functions into higher categories (KEGG pathways). The enrichment of predicted KEGG pathways was assessed with STAMP software (Parks et al., 2014) using a two-sided Welch's *t*-test with a Benjamini-Hochberg false discovery rate correction (*p* < 0.05).

### Quantitative Real-Time PCR for *TNF-α, FAS, KCNA3, NOD-2, SOD-2* Human Genes

For quantitative real-time PCR (RT-PCR), the DNA from biopsy samples was amplified and detected with a Stratagene Mx3000P Real-Time PCR System (Agilent Technologies Italia S.p.A., Milan, Italy) using the following Taqman gene expression assays: HS00174128_M1 (*TNF-*α, tumor necrosis factor-alpha), HS00236330_M1 (*FAS*, Fas cell surface death receptor), HS00704943_S1 (*KCNA3*, potassium voltage-gated channel subfamily A member 3), HS01550753_M1 (*NOD-2*, nucleotide-binding oligomerization domain containing 2), HS00167309_M1 (*SOD-2*, superoxide dismutase 2), and Hs999999 m1 (*GAPDH*, human glyceraldehyde-3-phosphate dehydrogenase). Human *GAPDH* was used as the housekeeping gene. PCR amplifications were carried out in a 20 μl total volume. In particular, the mixture reaction contained 10 μl of 2 × Premix Ex Taq (Takara, Japan), 1 μl of 20 × TaqMan gene expression assay, 0.4 μl of RoX Reference Dye II (Takara, Japan), 4.6 μl of water, and 4 μl of DNA. PCR conditions were 95°C for 30 s, followed by 40 cycles of 95°C for 5 s and 60°C for 20 s. PCR reactions were performed in duplicate using an MX3000p PCR machine (Stratagene, La Jolla, CA). The relative abundance of the expression of each gene was calculated by comparing delta cycle thresholds (Vigetti et al., 2008).

### Volatile Human Urinary Metabolome

A sensitive assay to identify volatile organic metabolites (VOMs) from urine was used to evaluate potential biomarkers. Urine was collected individually in safe, sterile boxes. A 20 ml glass vial was supplied with 2 g urine plus 10 μl of internal standard solution (2-pentanol-4-methyl) at 33 ppm. Vials were sealed with polytetrafluoroethylene-coated silicone rubber septa (20-mm diameter; Supelco, Bellefonte, PA, USA). To obtain the best extraction efficiency, the solid-phase microextraction (SPME) was performed by exposing a conditioned 75 μm Carboxen/PDMS fiber (Supelco, Bellefonte, PA, USA) to the headspace of 2 ml of acidified (pH 2) urine sample with 1 g of NaCl for 60 min at 60°C after a 35 min incubation (Semren et al., 2018). To keep the temperature constant during analysis, the vials were maintained on a heater plate (CTC Analytics, Zwingen, Switzerland) and the extraction was carried out with a CombiPAL system injector autosampler (CTC Analytics). The extracted compounds were desorbed in splitless for 3 min at 280°C. A Clarus 680 (PerkinElmer, Waltham, MA, USA) gas chromatograph equipped with an Elite-624Sil MS Capillary Column (30 m × 0.25 mm i.d., 1.4-μm film thickness; PerkinElmer) was used. The column temperature was set initially at 40°C for 3 min and then increased to 250°C at 5°C/min and to 280°C at 10°C/min and finally held for 5 min. Helium was used as the carrier gas at a flow rate of 1 ml/min. The analyses lasted 58 min. The single quadrupole mass spectrometer Clarus SQ 8C (Perkin Elmer) was coupled to the gas chromatography system. The source and transfer line temperatures were kept at 250 and 230°C, respectively. Electron ionization masses were recorded at 70 eV, and the mass-to-charge ratio interval was m/z 34 to 350. The GC-MS generated a chromatogram with peaks representing individual compounds. Each chromatogram was analyzed for peak identification using the National Institute of Standard and Technology 2008 (NIST) library. A peak area threshold >1,000,000 and 90% or a greater probability of matches was used for VOM identification, which was followed by manual visual inspection of the fragment patterns when required. Quantitative data for the compounds identified were obtained by the interpolation of the relative areas vs. the internal standard area.

### Statistical Analyses

Statistically significant differences in bacterial communities, comparing AA and healthy control samples, were detected by *t*-tests and one-way analysis of variance (ANOVA) tests for independent samples corrected by using the Tukey test. All datasets were normally distributed. Differences in human gene expression were assessed by the Wilcoxon-Mann-Whitney test. Analyses were performed with GraphPad Prism 7.0 (GraphPad Software, Inc., San Diego, CA). Differences between groups were considered significant at a *P* < 0.05. Correlations between the expression of human genes investigated by qRT-PCR and bacterial community compositions at the genus level (*p* < 0.05) were assessed based on Pearson's bivariate correlation and the results were graphically assessed using the corrplot R package.

### Availability of Data and Materials

16S sequences from both swab and biopsy samples of enrolled subjects obtained with the IlluminaSeq platform were deposited into the National Centre for Biotechnology Information (NCBI) BioProject database under the project number PRJNA510206.

## Results

### AA Affects the Microbiome Composition of the Scalp

Based on swab samples from AA subjects, a bacterial shift was found. At the species level, an increase in *Propionibacterium acnes* and a decrease in *Staphylococcus epidermidis* was previously reported (Pinto et al., 2019). In this work, by including three-layer biopsy samples, we found significant (*p* < 0.05) differences in the composition of the microbiome between healthy and AA subjects based on dermis and deep epidermis samples (Table 1). At the dermis level, *Candidatus Aquiluna* and two OTUs belonging to *Microthrixaceae* and ACK-M1 families showed significantly lower abundances in AA samples compared to those in healthy samples. In contrast, the *Acinetobacter* genus was associated with significantly higher abundances (*p* = 0.0493) in the dermis of the control group compared to those in AA subjects.

**Table 1:** Statistically significant differences in genera relative abundances between AA and healthy subjects (H) in deep epidermis (Deep ep.), dermis and hypodermis (Hyp).

**Genus**	**Deep ep. (AA)**	**Dermis (AA)**	**Hyp (AA)**	**Deep ep. (H)**	**Dermis (H)**	**Hyp (H)**	***P*-value deep ep**.	***P*-value dermis**	***P*-value hyp**
*Microthrixaceae* family *- genus unassigned*	0	0	0	0	0.23	0	–	**0.0035**	–
ACK-M1 family *- genus unassigned*	0.05	0.06	0.06	0	0.92	0	0.2707	**0.0438**	0.2707
*Candidatus Aquiluna*	0.75	0.35	0.27	0.18	2.38	1.19	0.29	**0.0486**	0.0666
*Staphylococcus*	10	13.22	6.48	49.58	48.36	10.21	**0.0421**	**0.0452**	0.3100
*SMB53*	0	0.06	0	0.41	0	0	**0.0416**	0.2707	−
*Neisseria*	1.31	1.40	1.67	0	0.23	0	**0.0324**	0.1727	0.0539
*Acinetobacter*	4.04	2.54	1.33	0	0	0.33	0.1371	**0.0493**	0.1769

*Only genera statistically significant (p < 0.05) in at least one layer have been reported*.

Regarding the epidermis layer, the genus SMB53 (family Clostridiaceae) was absent in AA samples, whereas *Anaerococcus* and *Neisseria* were relatively more abundant in the AA groups. The *Staphylococcus* genus was significantly less abundant in AA samples than in controls both in the epidermis and dermis (*p* = 0.0421 and 0.0452, respectively). At the species level, *Candidatus Aquiluna rubra* and *Staphylococcus epidermis* were significantly decreased in the dermis layer of AA samples compared to levels in healthy controls (*p* < 0.05), whereas an unassigned OTU at the species and genus levels, belonging to the *Neisseriaceae* family, was significantly more abundant in the epidermis of AA samples than in that of healthy subjects (*p* < 0.05). No statistically significant differences were found when comparing the hypodermis layers of AA and healthy subjects (data not shown). The Shannon index values were not indicative of differences in diversity among the analyzed biopsy layers (Table S1).

### Prediction of Metabolic Functions in the Skin Biopsy Layers

PiCRUST software and KEGG are useful tools to understand the biological features of the microbial population in a given ecosystem in terms of ecology and functional contributions (Langille et al., 2013). Although some differences in predicted functional profiles could distinguish AA and healthy samples, it was not possible to stratify biopsy layer samples based on total pathway prediction abundances. Specifically, healthy and AA samples generally clustered separately, but when looking for a per-layer grouping, the samples appeared to be mixed (data not shown).

**Figure 1:**
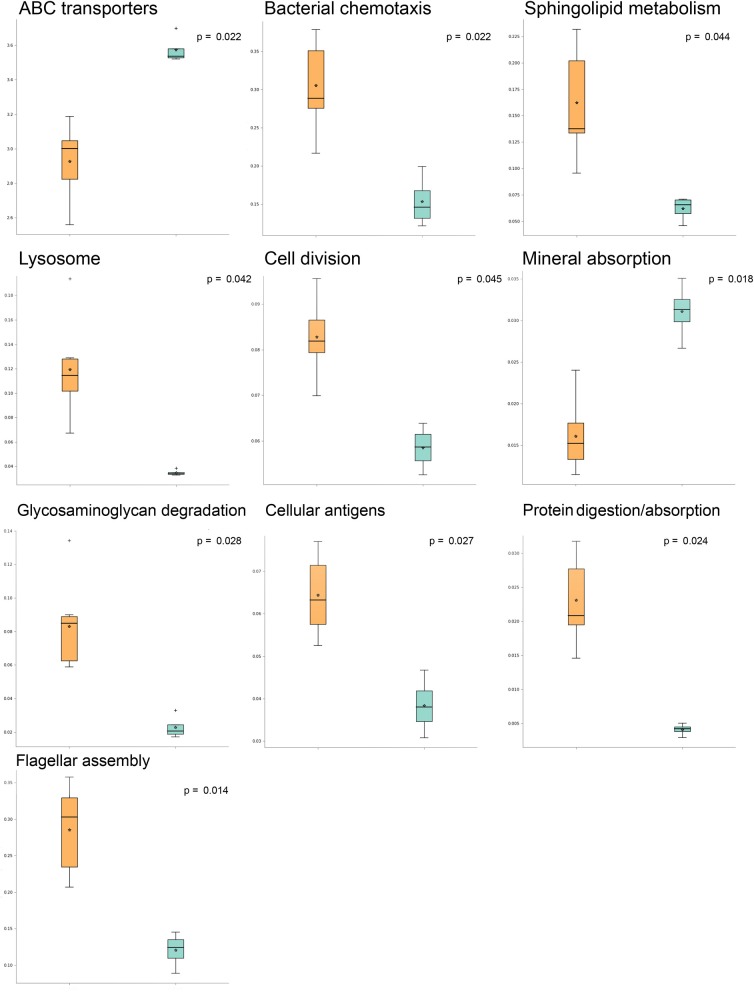
Significantly different pathways in alopecia areata (AA) vs. expression in healthy samples analyzed with STAMP (statistical analysis of taxonomic and functional profiles). Boxplots indicating pathway sample distribution (proportion of sequences) in AA (orange) and healthy (light blue) groups. Only statistically significant pathways are shown.

A comparison of predicted pathway abundances in dermis and hypodermis group layers vs. those in their healthy sample counterparts allowed us to identify a subset of 10 significantly different pathways in AA subjects. The list of significant pathways was derived from STAMP software and included flagellar, assembly, bacterial chemotaxis, mineral absorption, ABC transporters, cellular antigens, glycosaminoglycan degradation, lysosome, sphingolipid metabolism, cell division, protein digestion and absorption, and energy metabolism. The per-group boxplot distributions reported in Figure 1 show that predicted pathway abundances were significantly higher in AA than healthy samples. The only two exceptions were mineral absorption and ABC transporters. Pathway mean proportions and differences in mean proportions for each significant pathway are shown in Figure S1. No statistically significant differences (FDR > 0.05) in predicted pathways were found when comparing the AA set to both the epidermis and swab samples from healthy samples.

### AA Affects the Levels of *TNF-α, FAS, KCNA3, NOD-2*, and *SOD-2* Transcripts

**Figure 2:**
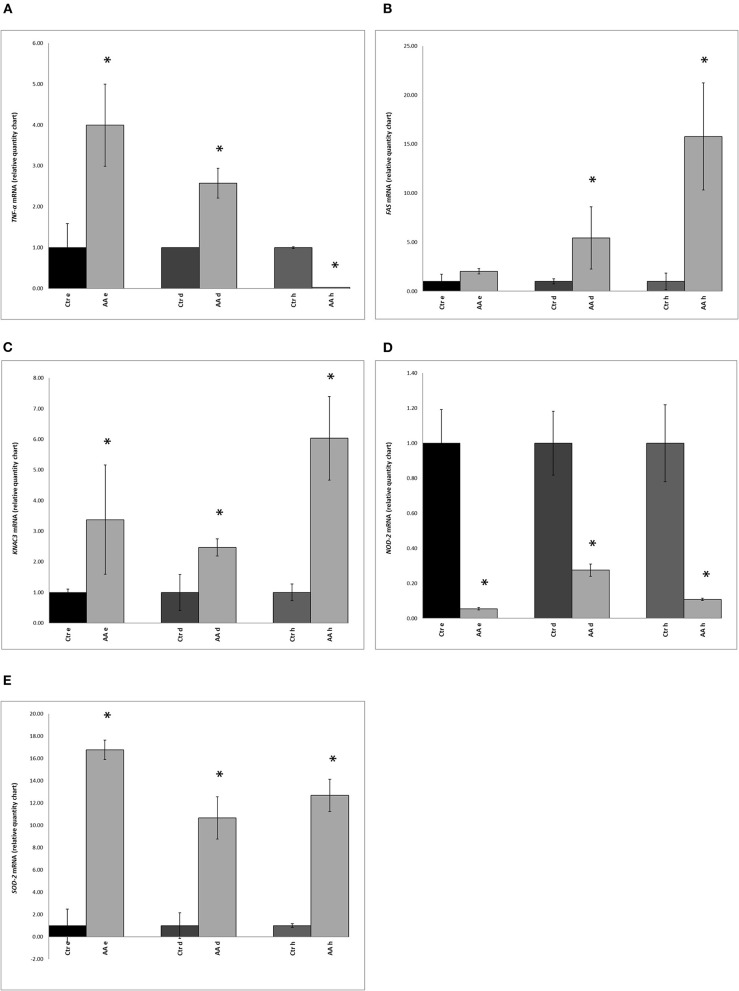
Expression of **(A)** *TNF-*α (tumor necrosis factor-alpha), **(B)** *FAS* (Fas cell surface death receptor), **(C)** *KCNA3* (potassium voltage-gated channel subfamily A member 3), **(D)** *NOD-2* (nucleotide-binding oligomerization domain containing 2), and **(E)** *SOD2* (superoxide dismutase 2) genes in biopsy samples, as determined by RT-PCR, in control (Ctr) and alopecia areata (AA) groups. Gene expression was measured in the deep epidermis (e), dermis (d), and hypodermis (h). Data represent the means ± SDs of three separate experiments performed in triplicate. Statistical differences between mean values were determined using the Wilcoxon-Mann- Whitney test. Asterisks indicate significant differences (*p* < 0.05).

With the aim of evaluating the expression of a subset of genes associated with inflammation and apoptosis in AA, we carried out RT-PCR analysis on *TNF-*α, *FAS, KCNA3, NOD-2*, and *SOD-2* genes. The expression of these five marker genes was determined in biopsy samples from the scalp of healthy and AA-affected individuals. Statistically significant (*p* < 0.05) differences were found in the expression of *TNF-*α between the control and AA-affected groups (Figure 2A). Compared to that in controls, higher expression of the *TNF-*α gene was reported in the deep epidermis and dermis of AA subjects (Figure 2A). No significant difference (*p* > 0.05) was found in the hypodermis biopsy layer between control and AA groups.

Compared to levels in healthy subjects, expression of the *FAS* gene was markedly different in the dermis and hypodermis of AA subjects (Figure 2B). A comparison of *KCNA3* gene expression between healthy subjects and the AA group also indicated significant differences. Moreover, the AA group showed the highest amount of *KCNA3* transcripts in all three layers and this difference was significant (Figure 2C). Further, the hypodermis and deep epidermis expressed higher levels of this gene than the dermis.

An opposite trend was observed for the *NOD-2* gene, with significantly lower expression in AA subjects than in healthy samples (Figure 2D). *SOD-2* gene expression was found to be significantly higher in the AA group than in the control group (Figure 2E). Further, upon comparing the three layers, the highest *SOD-2* expression level was found in the deep epidermis of the AA group. Among AA subjects, the expression of *SOD-2* was higher (*p* < 0.05) in the deep epidermis and hypodermis layers than the dermis.

### Statistically Significant Correlations Between Microbiome and *TNF-α, FAS, KCNA3, NOD-2*, and *SOD-2* Transcripts

**Figure 3:**
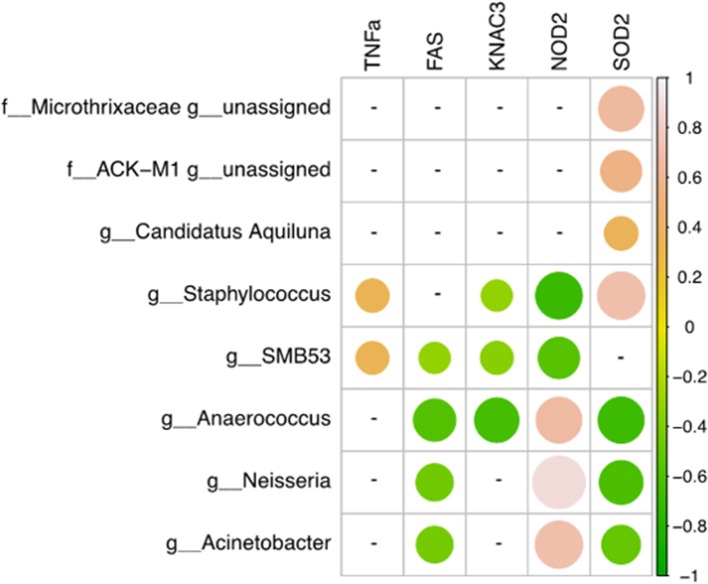
Correlations between human gene expression levels and genera. Pearson correlation values were calculated between human gene expression levels (qRT-PCR) and the percent abundance of genera. The R corrplot package was used to visualize the correlation matrix, which was then reordered using the hclust method in R. The correlation coefficient scale ranges from −1 (light pink for negative correlations) to 1 (green for positive correlations). Only statistically significant correlation values (*p* < 0.05) and with a correlation coefficient |0.3 < r < 1| were plotted.

A Pearson's correlation analysis was used to determine the relationships between human gene expression levels analyzed by qRT-PCR and microbiome composition and only statistically significant correlations (*p* < 0.05) were shown (Figure 3). The relative mRNA expression of *FAS* and *SOD2* genes was negatively correlated with *Anaerococcus, Neisseria*, and *Acinetobacter* genera. In contrast, *NOD2* was positively correlated with these three genera but negatively associated with *Staphylococcus* and *SMB53. KCNA3* was negatively correlated with *Anaerococcus, SMB53*, and *Staphylococcus*. Finally, *SOD2* gene expression was negatively correlated with *Anaerococcus, Neisseria*, and *Acinetobacter* and positively associated with *Staphylococcus, Candidatus Aquiluna*, and two unassigned genera belonging to the Microthrixaceae and ACK-M1 families.

### Characterization and Comparative Analysis of Urinary Volatile Metabolites

An objective comparison of metabolomic patterns found in urine from AA patients and healthy volunteers was established based on qualitative and quantitative differences in VOMs using HS-SPMECAR/PDMS/GC–MS methodology. Sixty-two volatile metabolites, found in the urine of both AA and healthy subjects, included a variety of chemical structures that are potentially involved in several biological functions. VOMs were identified from urine samples and grouped according to chemical classes as follows: alcohols (4), aldehydes (4), carboxylic acid (7), esters (3), ketones (10), sulfur compounds (4), phenols (5), terpenoids (12), furanic compounds (7), and other aromatic compounds (6) (data not shown). Compared to those in healthy subjects, some compounds (menthol, methanethiol, dihydrodehydro-beta-ionone, 2,5-dimethylfuran, 1,2,3,4, tetrahydro-1,5,7-trimethylnapthalene) were found at significantly (*p* < 0.05) higher levels in the urine of AA patients compared to that in healthy subjects (Table 2).

**Table 2:** Concentrations of VOMs (μg/g) in urine samples detected in alopecia areata patients compared to healthy subjects.

	**Healthy**	**AA**	**Healthy vs. AA**
	**μg/g**	**μg/g**	***p*-values**
Methanethiol	0.029	0.066	0.041
2,5-Dimethylfuran	0.010	0.019	0.021
Menthol	0.000	0.007	0.035
Dehydro-beta-ionone	0.002	0.027	0.001
1,2,3,4,Tetrahydro-1,5,7-trimethyl napthalene	0.117	0.416	0.030

*Only statistically significant differences (Welch test) were reported*.

## Discussion

By analyzing three biopsy layers, specifically the deep epidermis, dermis, and hypodermis, in the current study, we highlighted the differences in microbial populations inhabiting the scalp of AA subjects compared to those in a healthy cohort. The significant differences in genera abundances (*p* < 0.05) were found in the hypodermis and especially in the dermis layer. These findings are consistent with the results of our previous study, where we reported the presence of a bacterial shift on the scalp of subjects suffering from AA (Pinto et al., 2019). Furthermore, this effect was found to be mainly exerted by *Propionibacterium* and *Staphylococcus* genera among AA subject microbiomes, a finding that was also reported in other studies (Wang et al., 2012; Juhász and Atanaskova Mesinkovska, 2019). A decrease in some species such as *Candidatus Aquiluna rubra* and *Staphylococcus epidermidis* and an increase in the *Neisseria* genus at the dermis level revealed specific characteristics associated with AA. Interestingly, *Candidatus Aquiluna rubra*, a marine member of the *Actinobacteria* class, was previously found to be considerably less abundant in breast cancer patients than in healthy controls (Parida and Sharma, 2019). We also found an increase in gram-positive anaerobic cocci such as *Anaerococcus*. Bacteria of this genus stimulate rapid induction of antimicrobial peptide responses in human keratinocytes, which could be an important signaling mechanism associated with keratinocytes when the skin is injured (Fyhrquist et al., 2019). Differences in the microbiota composition, especially in the dermis layer, are in line with a recent work from Bay and collaborators (Bay et al., 2020) in which, for the first time, they reported the concept of a dermal “core” microbiome. In particular, they demonstrated the similarity among individuals in dermal microbiome and that it is derived from a specific subset of the epidermal microbiota. Starting with 16S sequencing data, we explored the differences in predicted KEGG pathways between AA and healthy samples. Some KEGG pathways were mainly associated with AA samples. Among these, environmental information processing (bacterial chemotaxis and flagellar assembly) was predominant in AA samples. Bacterial chemotaxis is reported to promote activated T lymphocytes in autoimmune disorders including AA (Abdolmaleki et al., 2018). Moreover, chemotaxis permits bacterial species to access particular host niches, which allows the bacteria to express or deliver proapoptotic host cell factors (Rolig et al., 2011). The analyses of KEGG predicted pathways also showed a significant increase in the cellular antigens pathway in AA samples. AA is recognized as a cell-mediated autoimmune hair loss disease, and associated autoantigen epitopes have been recently identified (Wang et al., 2016). Moreover, microbial-derived antigens were reported to be responsible for the susceptibility of the follicle to an autoimmune attack (Paus et al., 1993). These molecules appear to act by enhancing MHC I expression and in a minority of patients, also by affecting MHC-non-restricted polyclonal activators of CD8+ T cells (Mueller et al., 2014; Roitt et al., 1993).

We contextually detected a significant difference in predicted pathways associated with glycosaminoglycan (GAG) degradation in AA samples. From a clinical perspective, alterations of GAG degradation induce mucopolysaccharidoses and abnormalities in hair morphology (Malinowska et al., 2008), and studies in patients with hair loss revealed a reduction in GAGs in the connective tissue around the follicle, suggesting their importance for structure and density maintenance (Maniatopoulou et al., 2016). According to Cole and collaborators (Cole et al., 2011), bacteria can use GAGs, especially hyaluronan, to mask themselves, avoiding recognition by the immune system. Indeed, some pathogenic bacteria target GAGs to colonize the skin. In AA samples, the increase in GAG degradation pathways corresponds to a significant decrease in ABC transporters, which are responsible for the import of host mammalian GAGs (Oiki et al., 2017).

Moreover, modifications in the metabolism of micro- and macronutrients (Thompson et al., 2017; Gade et al., 2018; Ruiz-Tagle et al., 2018; Almohanna et al., 2019) play a pivotal role in the development of AA, and the resident microbiota can itself contribute to nutrient synthesis. As reported (Thompson et al., 2017), the pathophysiological mechanism through which sub-threshold levels of micronutrients might contribute to AA include the dysregulation of immune cell function, dysregulation of coenzyme-dependent enzyme function in DNA synthesis, and an imbalance between oxidant and antioxidant activity. The pathway relative to mineral absorption and ABC transporters was predicted to be significantly lower in AA samples. This reduction might imply a further risk associated with the development of AA. A significant increase in sphingolipid metabolism in the AA group was also found compared to that in healthy subjects. Numerous biological functions are attributed to sphingolipids (Chatterjee and Pandey, 2008) and recently, a role for sphingolipid metabolism in hair growth via inflammation was reported (Bedja et al., 2018).

Microorganisms typically inhabiting the superficial epidermis can get access to keratinocytes of the scalp and interact with the cutaneous immune system (Saunders et al., 2006). As a consequence, they can influence the immunological status of the scalp and hair follicles in response to external and internal stress (Naik et al., 2012; Scharschmidt, 2017). The microbiome can also affect scalp metabolic activity and the transcriptomic framework. Metabolic intermediates in the cholesterol biosynthetic pathway directly influence signaling pathways relevant to immune responses and hair growth, including JAK-signal transducer and activator of transcription and peroxisome proliferator-activated receptor pathways (Renert-Yuval and Guttman-Yassky, 2017).

AA is characterized by a strong inflammation (Wolff et al., 2016) taking place in the upper third of the hair follicle, a region that harbors a large number of microorganisms. Therefore, AA subjects exhibit a disequilibrium in inflammatory cytokines (Bodemer et al., 2000); the production of these molecules in skin and scalp can also be regulated by metabolites produced by microorganisms (Watanabe et al., 2001; Qin and Wade, 2018). Various studies have established AA as a T-cell-mediated autoimmune disease and multiple cytokines, especially the helper T-cell type 1 cytokines such as TNF-α, are required for the inflammation process. Kasumagic-Halilovic and collaborators (Kasumagic-Halilovic et al., 2011) reported that the proinflammatory cytokine TNF-α is a key player in the development of AA. In line with these findings, we found an increase in *TNF-*α gene expression, especially in the deep epidermis and dermis of AA subjects.

The abnormal expression of FAS has also been reported (Bodemer et al., 2000). This protein is a key mediator of the apoptotic process involving the dermal papilla cells of the hair follicle of AA subjects (Norris et al., 1995; Bodemer et al., 2000). We detected an increase in *FAS* gene levels with increasing depth of biopsy layers.

In several autoimmune disorders, including AA, activated T lymphocytes also express a variety of potassium channels, among which KCNA3 (Gilhar et al., 2013) was found to be overexpressed in the sub-epidermal compartments of the scalp of AA subjects analyzed in this study. As previously shown, AA has a strong immunological component (Odom et al., 2006). Among effectors of the cellular immunological response, *NOD2* plays a crucial role in maintaining the balance between immune responses of the microbiome and the host (Negroni et al., 2018). Further, commensal bacteria can promote *NOD2* expression via a negative feedback mechanism.

Another important player in the etiopathology of AA is oxidative stress (McElwee et al., 2013). In AA patients, oxidative stress occurs as a consequence of the overproduction of free radicals and inadequate antioxidant defense. The microbiota is reported to play a role in the self-defense mechanism of the skin against oxidative stress. Prie and collaborators (Prie et al., 2015) reported the overproduction of enzymes involved in oxidative stress, such as SOD2, in patients affected by AA. This is in line with the increase in the expression of the *SOD2* gene observed in the AA group, as compared to that in the healthy group in this study.

The correlation between microbial composition at the genus level and gene expression allowed us to highlight the connections among inflammation, immune responses, and other dysbiotic conditions in AA subjects. Although our number of samples was low and the intimate interactions between skin microbiota and host cells remain unknown, our data showed that some genera are positively correlated with *SOD2* and *NOD2* human transcripts. The dysbiosis occurring in AA patients was also found to be associated with a specific signature in terms of genera. *Anaerococcus, Neisseria*, and *Acinetobacter* were significantly more abundant in AA subjects and negatively correlated with *FAS* and *SOD2* genes but were positively correlated with *NOD2*.

By inspecting VOM profiles, we also detected statistically significant differences between AA and healthy subjects. Of minor importance, menthol and dehydro-beta-ionone metabolites are used as skin lotion topical treatment excipients and/or ingredients, and thus, these products are probably the main source of these metabolites found in urine. 1,2,3,4,Tetrahydro-1,5,7-trimethyl naphthalene was detected at a higher level in AA subjects. Evidence suggests that naphthalene derivatives might be a result of steroid degradation (Silva et al., 2012).

Intralesional corticosteroids are the treatment of choice for adults with AA (Alkhalifah et al., 2010); however, topical corticosteroids used in common commercialized shampoo might also lead to the persistence of these volatile sulfur compounds (VSCs) in urine. Another VSC, namely methanethiol (MeSH), derived from methionine metabolism, was found to be significantly higher in AA samples than in controls; associated toxicity has been detected in patients with homocystinuria caused by cystathionine β-synthase deficiency, but this compound has also been reported to be present in the urine of humans after eating asparagus (Tangerman, 2009).

Some studies suggest that bacteria convert dimethylsulfoniopropionate (DMSP) into volatile sulfur compounds such as dimethylsulfide and MeSH (Tangerman, 2009). Of note, MeSH is an important precursor of methionine and protein sulfur and some genera belonging to *Actinobacteria* can metabolize it and use it for assimilatory purposes; for example, marine bacterioplankton incorporate MeSH directly into methionine via the action of cystathionine γ-synthetase (Kiene et al., 1999). Although Kiene and colleagues focused exclusively on marine microorganisms, their findings shed light on sulfur acquisition by freshwater microorganisms as well. Whereas, DMSP appears to be restricted primarily to marine habitats, MeSH is present in all aquatic environments. Dissimilatory SO${}_{4}^{2-}$ reduction by sulfate-reducing bacteria occurs in anoxic marine sediments or in freshwater environments, where these organisms use several electron donors such as hydrogen and various organic compounds such as ethanol, formate, lactate, pyruvate, fatty acids, methanol, and methanethiol (Muyzer and Stams, 2008).

In AA subjects, we found a statistically significant difference in the relative abundance of *Candidatus Aquiluna rubra* species. This species belongs to the actinobacterial clade and is also present in freshwaters (Kang et al., 2012). We hypothesized it might contribute to methanethiol metabolism in the hypoxic environment of the dermis. Moreover, we found a significant difference in the 2,5-dimethylfuran (DMF) metabolite. DMF is one of the main metabolites of hexane in humans. It plays a role in the neurotoxicity of hexane, has been identified as one of the components of cigar smoke, and is a marker of tobacco smoke exposure (Hecht, 2003). Considering that our samples belonged to a non-smoking AA cohort, we hypothesize that DMF, as another derivative furan compound (Hecht, 2003), has an important role in quorum sensing processes through which bacteria monitor population density in AA bald spots. Since the correlative and cross-sectional nature of the study, further analyses and collateral approaches will lead to a more complete examination of AA pathophysiology.

## Conclusions

Studies on the microbiome of the deeper portions of the scalp are scarce. Using a 16S metagenetic approach, we inspected the composition of the scalp microbiome in AA patients and highlighted how some genes related to the inflammation process significantly differ from those of healthy samples. Starting from 16S rRNA and in silico analysis, we identified some significant differences in predicted pathways related to AA pathologic conditions. Further, for the first time, we also detected some VOMs that distinguish AA subjects. This multiple comparison approach highlights the potential traits associated with AA. High throughput microbiome sequencing will be of fundamental importance to understand the AA microbiome and will help to elucidate scalp–commensal bacteria interactions.

## Data Availability Statement

The datasets generated for this study can be found in the PRJNA510206.

## Ethics Statement

The studies involving human participants were reviewed and approved by Ethical Independent Committee for Clinical, not pharmacological investigation in Genoa (Italy). The patients/participants provided their written informed consent to participate in this study.

## Author Contributions

MD, FR, DP, FMC conceptualized the study. DP and FMC contributed to methodology. DP, FMC, and GC worked on the formal analysis. FR, MD, DP, FMC, and GC carried out the investigation. DP and FMC worked on the software. DP and FMC wrote the original draft. FR, MD, DP, FMC, and GG reviewed and edited the manuscript. FR, MD, and MG supervised the study. FR, MG, and GG acquired the funding.

## Conflict of Interest

DP is employed by Giuliani S.p.A. FR and MG serve as a consultant for Giuliani S.p.A. GG is member of Board of Direction. The remaining authors declare that the research was conducted in the absence of any commercial or financial relationships that could be construed as a potential conflict of interest.

## Supplementary Material

Figure S1 Mean values (mean proportion, %) and STAMP extended error bar relative to the ten statistically significant pathways in AA and healthy sample comparison. Extended error bar plot for all FDR statistically significant pathways between the dermis microbiome of AA (orange) compared to healthy (light blue) groups. P-values have been obtained two tailed Welch's t-test (corrected by using Benjamini-Hochberg FDR).
