# Supplementary figures and images for: Predictive Metagenomic Profiling, Urine Metabolomics, and Human Marker Gene Expression as an Integrated Approach to Study Alopecia Areata
Source: Front Cell Infect Microbiol. 2020 Apr 29;10:146. doi: 10.3389/fcimb.2020.00146 (PMC7201066; doi:10.3389/fcimb.2020.00146)

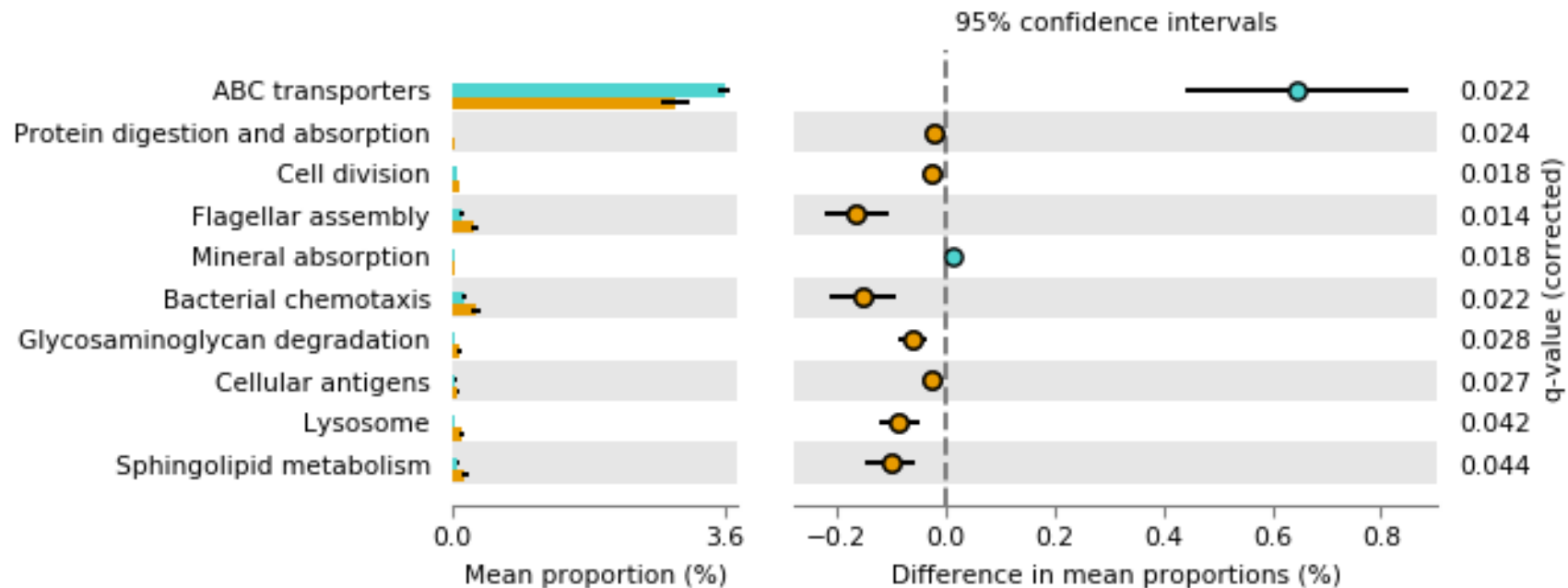

Supplement: Figure S1 — Mean values (mean proportion, %) and STAMP extended error bar relative to the ten statistically significant pathways in AA and healthy sample comparison. Extended error bar plot for all FDR statistically significant pathways between the dermis microbiome of AA (orange) compared to healthy (light blue) groups. P-values have been obtained two tailed Welch's t-test (corrected by using Benjamini-Hochberg FDR). [file Data_Sheet_1.PDF]
